# Chemical and biological assessment of metal organic frameworks (MOFs) in pulmonary cells and in an acute in vivo model: relevance to pulmonary arterial hypertension therapy

**DOI:** 10.1177/2045893217710224

**Published:** 2017-06-27

**Authors:** Nura A. Mohamed, Robert P. Davies, Paul D. Lickiss, Blerina Ahmetaj-Shala, Daniel M. Reed, Hime H. Gashaw, Hira Saleem, Gemma R. Freeman, Peter M. George, Stephen J. Wort, Daniel Morales-Cano, Bianca Barreira, Teresa D. Tetley, Adrian H. Chester, Magdi H. Yacoub, Nicholas S. Kirkby, Laura Moreno, Jane A. Mitchell

**Affiliations:** 1 Department of Cardiothoracic Pharmacology, National Heart and Lung Institute, Imperial College, London, UK; 2 Heart Science Centre at Harefield Hospital, Harefield, UK; 3 Qatar Foundation Research and Development Division, Doha, Qatar; 4 Department of Chemistry, South Kensington Campus, Imperial College, London, UK; 5 Department of Pharmacology, Faculty of Medicine, Universidad Complutense de Madrid- Instituto de Investigacion Sanitaria Gregorio Marañón (IiSGM), Ciber Enfermedades Respiratorias (CIBERES), Spain; 6 Lung Cell Biology Group, National Heart and Lung Institute, Imperial College London, London, UK

**Keywords:** nanoparticles, nanotechnology, MIL-89, MIL-89 PEG, endothelial cells, vascular smooth muscle cells

## Abstract

Pulmonary arterial hypertension (PAH) is a progressive and debilitating condition. Despite promoting vasodilation, current drugs have a therapeutic window within which they are limited by systemic side effects. Nanomedicine uses nanoparticles to improve drug delivery and/or reduce side effects. We hypothesize that this approach could be used to deliver PAH drugs avoiding the systemic circulation. Here we report the use of iron metal organic framework (MOF) MIL-89 and PEGylated MIL-89 (MIL-89 PEG) as suitable carriers for PAH drugs. We assessed their effects on viability and inflammatory responses in a wide range of lung cells including endothelial cells grown from blood of donors with/without PAH. Both MOFs conformed to the predicted structures with MIL-89 PEG being more stable at room temperature. At concentrations up to 10 or 30 µg/mL, toxicity was only seen in pulmonary artery smooth muscle cells where both MOFs reduced cell viability and CXCL8 release. In endothelial cells from both control donors and PAH patients, both preparations inhibited the release of CXCL8 and endothelin-1 and in macrophages inhibited inducible nitric oxide synthase activity. Finally, MIL-89 was well-tolerated and accumulated in the rat lungs when given in vivo. Thus, the prototypes MIL-89 and MIL-89 PEG with core capacity suitable to accommodate PAH drugs are relatively non-toxic and may have the added advantage of being anti-inflammatory and reducing the release of endothelin-1. These data are consistent with the idea that these materials may not only be useful as drug carriers in PAH but also offer some therapeutic benefit in their own right.

## Introduction

Pulmonary arterial hypertension (PAH) is a rare yet severe disease characterized by increased pulmonary artery pressure (PAP) leading to augmented workload on the right side of the heart. If not treated, this ultimately results in heart failure and death.^1^ The pathology of PAH is characterized by proliferation of pulmonary artery vascular smooth muscle cells (PAVSMCs) within the vessel wall leading to vascular remodeling in small vessels resulting in loss of lower vessel mass. In more severe cases, clonal endothelial lesions, known as “plexiforms,” which consist of highly proliferative endothelial cells can also develop in the lungs.^2–4^ A deregulation of vasoactive mediators released by endothelial cells in pulmonary arteries underpins PAH, with elevated levels of the constrictor peptide, endothelin (ET)-1 being a key therapeutic target in the treatment of the disease. In addition, PAH is now established as an inflammatory disease associated with increased levels of cytokines, such as CXCL8 and interferons.^5^

Current PAH therapies are based on manipulating or mimicking endogenous vascular hormones that maintain homeostasis.^6^ These therapies are categorized into four classes of dilator drugs, each targeting a specific endogenous pathway: (1) prostacyclin drugs;^7,8^ (2) phosphodiesterase type 5 inhibitors;^9,10^ (3) ET receptor antagonists;^11,12^ and most recently (4) soluble guanylate cyclase activators^13^ (Supplementary Table 1). However, these therapies are all fundamentally limited by systemic side effects, which limit the concentration of drug that can be used. This point is illustrated by the findings in some preclinical in vivo studies,^14^ where PAH drugs, given at many times the dose used in man, actually reverse disease in laboratory animals. This raises the idea that current PAH drugs could work better, even reverse disease, if they were to be delivered at higher doses directly to the affected vessels. PAH is therefore a prime candidate disease for targeted drug delivery directly to the affected pulmonary vessels. In other diseases, such as cancer, this has been achieved using nanomedicine technology. We^15^ and others^16^ therefore suggest that targeted drug delivery using nanotechnology will overcome the limitations of current drugs and, we suggest, convert this fatal disease into a chronic treatable condition.

Nanotechnology, applied in engineering, chemistry, or medicine, is defined as the ability to use structures less than 100 nm to improve the properties and/or functions of compounds.^17,18^ Several advantages in applying nanomedicine in the treatment of PAH exist including: (1) targeted delivery, avoiding systemic side effects; (2) controlled and sustained drug release avoiding the need for continued infusions; and (3) increased drug permeability and absorption which helps to maintain a homogenous drug distribution in tissues.^19^

There are several types of nanoparticles that can be considered as carriers for PAH drugs. These include: (1) liposomes; (2) micelles; (3) polymeric nanoparticles; (4) nanocrystals and nanoprecipitates; (5) metal organic frameworks (MOFs); (6) metal-organic polyhedra (MOPs); and (7) inorganic nanoparticles such as gold clusters. Our recent work has described a novel nanomedicine formulation consisting of a nitrated polymer that releases nitric oxide (NO) slowly in aqueous solution.^20^ However, this molecule, while suitable as a vasodilator and potential adjunct therapy, is limited to the NO pathway. Our current study focuses on MOFs, which are microporous crystalline materials, as potential carriers of all currently used PAH drugs. A number of MOF materials from the MIL (Materials of Institute Lavoisier) family, namely MIL-89, MIL100, etc.^21–25^ have shown particular promise for applications as drug delivery platforms. This is because they: (1) can be prepared in small (nano) particulate sizes; (2) are biocompatible and biodegradable;^26^ (3) have a large internal surface area and low density with commensurate high drug loading capacity; (4) have reasonable thermal and mechanical stability; and (5) have a long drug release period with the ability to incorporate different functional groups.^21–24^

Of particular interest is the nanoMOF MIL-89 and its PEGylated form MIL-89 PEG, which consist of iron-based clusters as the metal-based building unit and *trans-trans* muconic acid as the organic linking unit. MIL-89 PEG differs from MIL-89 by addition of a alpha-methoxy-omega-amino poly(ethylene glycol) (PEG-MW 5000 Da) coating on the surface of the MIL-89 nanoparticle, which allows the formation of a more uniformed nanoparticle structure and prolongs the half-life of the nanoparticle. MIL-89 and MIL-89 PEG can be prepared with a particle size of 50–100 nm and have been shown to accommodate the anti-cancer drug busulfan and the anti-viral drug cidofovir.^24^ Based on the calculated molecular dimensions of busulfan and cidofovir, all of the current PAH drugs are theoretically capable of fitting within the channels of the MOF, with the smallest two quoted dimensions less than the cross-section of the channels (Supplementary Table 1). Moreover, a significant advantage of iron based MOFs, such as MIL-89, is that they can be used as contrast agents for in vivo imaging using magnetic resonance imaging^24^ allowing both the tracking of drug distribution and progression of disease.

However, the effects of iron-based MOFs, such as MIL-89, on functions of cells relevant to PAH are not known. Thus, as a critical prelude to taking iron based MOF formulations forward into PAH drug therapy, here we investigated the influence of MIL-89 and MIL-89 PEG on the viability and mediator release from a range of cell lines including vascular cells cultured from patients with PAH and tested the effects of MIL-89 on a range of toxicological readouts in rats dosed for up to 14 days.

## Methods

### Preparation of MIL-89

MIL-89 was prepared as previously described.^17–26^ Briefly, iron(III) chloride hexahydrate (FeCl_3_.6H_2_O) (MW = 270.3; 1 mmol; Sigma Aldrich®, UK) and *trans-trans* muconic acid (MW = 142.1; 1 mmol; Sigma Aldrich®, UK) were mixed in 10 mL of absolute ethanol (99.5%; Sigma Aldrich®-UK), heated at 100℃ for 15 h in a Parr reactor and the precipitate recovered by centrifugation at 10,500 rpm for 15 min. The sample was purified by serial washes in deionized water and air dried to retrieve the brown precipitate of MIL-89 (10 mg), which was used in further studies.

The PEGylated form of MIL-89 (MIL-89 PEG) was prepared as above with the following modifications; FeCl_3_.6H_2_O (MW = 270.3; 1 mmol; Sigma Aldrich®, UK), *trans-trans* muconic acid (MW = 142.1; 1 mmol; Sigma Aldrich®, UK) and alpha-methoxy-omega-amino poly(ethylene glycol) (PEG-MW 5.000 Da; IRIS Biotech-Germany) were dissolved in 10 mL of absolute ethanol (99.5%; Sigma Aldrich®, UK), heated to 100℃ for 6 h and centrifuged to retrieve the creamy color precipitate. The sample was washed with deionized water, air-dried, and ground to a fine powder (50 mg) for use in further studies (see below).

### Chemical analysis of MOFs

The characterization and purity of the target MOFs was primarily assessed using powder X-ray diffraction studies. For both MOFs, MIL-89 and MIL-89 PEG, the number and position of the peaks in the diffraction patterns corresponded directly to literature reported values for these materials.^27,28^ In addition, infrared/attenuated total reflection (IR/ATR) spectroscopic studies were also in agreement with literature reports.^27,28^ Thermogravimetric analysis was undertaken on all samples. Scanning electron microscopy (SEM) was used to determine the particulate size of the MOFs with image data analyzed using Image J Software.^29^

### Cell lines

Here, using standard culture techniques we have described previously, the following cell types were obtained: (1) endothelial cells grown from human blood of either control donors^30^ or patients with PAH;^31^ (2) human pulmonary artery endothelial cells (PAECs; cultured according to suppliers recommendations (PromoCell; UK); (3) human PAVSMCs,^31^ human airway smooth muscle cells (HASMCs),^32^ human type II pneumocytes (A549 cells),^33^ and murine J774 macrophages.^34^

### Effects of MIL-89 and MIL-89 PEG cells in vitro

Endothelial and PAECs were plated at a seeding density of 10,000 cells/well whereas PAVSMCs, HASMCs, and A549 and J774 cells were plated at a seeding density of 100,000 cells/well in 96-well plates, respectively. After plating, all cell types were incubated overnight before being treated with MIL-89, MIL-89 PEG, or the starting materials for MIL-89 (muconic acid, PEG, or FeCl_3_.6H_2_O). In some studies, to induce inflammatory responses, cells were treated with LPS (1 µg/mL) (Sigma-Aldrich, UK) for 24 h. Conditioned media was collected at the end of the incubation period for the measurement of NO by nitrite using the Griess assay as we have described previously^34^ or CXCL8 and ET-1 both by specific enzyme-linked immune assay (ELISA; R&D Systems) or LDH (Abcam, UK) according to manufacturer’s instructions. Media was then replaced with AlamarBlue® (Life Technologies, UK) solution for the measurement of cell viability. In some experiments cell number was assessed by imaging using a Cellomics VTi HCS Arrayscanner (Thermo Fisher, Pittsburgh, PA, US) as we have done previously.^35^ Cells were stained with 4',6-diamidino-2-phenylindole (DAPI) and the following parameters assessed: (1) the number of cells in each well (cell/field); (2) the number of cells in the whole well (cell/well); and (3) changes in nuclear shape of the cells in the whole well (mean). Cells treated with 10 mM H_2_O_2_ were used as negative control (100% dead) in each single experiment. In separate experiments assessment of apoptosis and necrosis were made using Abcam’s Annexin V-FITC Apoptosis Detection Kit (Reference: ab14085) according to the manufacturer’s instructions.

### In vivo assessment of chronic administration of MIL-89

All animal work conformed to the Directive 2010/63/EU of the European Parliament and the procedures were approved by the institutional Ethical Committee (Comité de Experimentación Animal de la Universidad Complutense de Madrid). Male Wistar rats (250 g body weight; Harlan Iberica) were randomly allocated into the following experimental groups: control (n = 22) and MIL-89 (n = 20). Rats were treated with vehicle (saline solution; 0.9% NaCl) or MIL-89 (50 mg/kg/day) by intraperitoneal injection (i.p.) twice a week for up to two weeks. We selected the dose of 50 mg/kg/day based on safety data reported in a similar study by Baati et al.^36^ using the related iron MOFs MIL-88 and MIL-100. In the Baati study, single one-off doses of 30, 50, 110, or 220 mg/kg were administered to rats and animals followed for up to 30 days with no long-lasting toxic effects.^36^ In order to include a cumulative-dose approach we opted for a protocol where single doses and multiple doses could be compared. It should also be noted that the i.p. route of administration for MIL-89 was selected in this initial study rather than a more direct, i.v. route for the following two reasons: (1) i.v. administration iron-based nanoparticles are rapidly cleared from the circulation^37,38^ whereas when administered i.p. levels in the blood increase more slowly and remain constant for longer periods of times;^37^ and (2) i.p. injection of nanoparticles limit accumulation in the liver, thereby limiting their potential side effects in this organ. Body weight and animal behavior were monitored every day. Two animals per group were killed at the following time points: single dose on days 1 and 3 and multiple doses on days 7, 10, and 14; data were pooled from animals at days 1–3 and at days 7, 10, and 14. Plasma samples and organs (lung, heart, liver, spleen, kidneys, thymus, and brain) were collected, weighed, snap frozen, and kept at –80℃ until further analysis. One of the left lung lobes was weighed and dried in an oven at 50℃ for 24 h in order to determine pulmonary edema by calculating the wet to dry lung weight ratio.

### Lung histology

The right lung was inflated in situ with 10 mL of 4% paraformaldehyde (PFA), removed, and embedded in paraffin for sectioning. To visualize iron deposits, lung sections (5 µm) were stained with Prussian blue using the Accustain® Iron Stain kit (Sigma-Aldrich).^39^ Sections were examined by light microscopy and the number of iron particles was counted in a blinded fashion. In some sections, a double staining of von Willebrand Factor (vWF) and Prussian blue was conducted following antigen retrieval in sodium citrate buffer.^40^ Staining of endothelial cells was performed using a rabbit anti-vWF antibody (1:200 dilution) and developed following the manufactures’ instructions (Chemicon Blood Vessel Staining Kit ECM590). Once the desired color intensity was reached, the slides were subjected to the Prussian blue staining protocol described above.

### Measuring oxidative stress

Oxidative stress in plasma samples was determined by measuring the static and capacity oxidation-reduction potential (ORP) using the RedoxSYS™ diagnostic system (Colorado, USA), following manufacturer’s instructions.

### Markers for organ failure

Circulating levels of proteins (total protein, albumin, globulin, and AG ratio) and the following markers were measured in plasma by Catalyst Dx® Chemistry Analyzer (IDEXX Laboratories; UK): urea, creatinine, alanine transaminase-ALT, alkaline phosphatase-ALK, gamma glutamyl transferase-GGT), total bilirubin, cholesterol, inorganic phosphorus, calcium, and glucose.

### Measuring iron levels in plasma and tissue homogenates

Iron levels in plasma and tissue homogenates were measured using the ferrozine reagent as described previously.^39^ Iron (0–128 µg/mL) and MIL-89 (0–1280 µg/mL) were used as standards.

### Statistical analysis

Data are the mean ± SEM and statistical significance (taken as *P* < 0.05) was determined using GraphPad Prism 5 as described in each figure legend.

### Ethics statement

Blood was collected for this study under ethical approval from the National Institute of Health Research. Informed consent was acquired for collection of human blood from control volunteers (NRES reference 08/H0708/69) and patients with PAH (NRES reference 10/H0504/9).

## Results

### Chemical characterization of MIL-89 and MIL-89 PEG

**Fig. 1.**
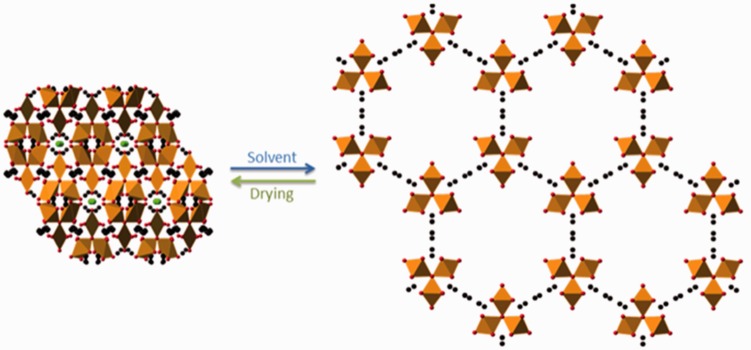
Predicted 3D structure of hydrated and dehydrated MIL-89. Images were obtained using CrystalMaker software 9.2 and data from Serre et al.^27^ The structures are “viewed” down the z-axis. The orange polyhedra represents iron atoms while red, black, and green spheres represent oxygen, carbon, and chlorine atoms, respectively.

Preparation of the MOFs MIL-89 and MIL-89 PEG was undertaken as described previously^24^ and the chemical composition and structure verified using IR/ATR, powder X-ray diffraction, and SEM, respectively.^27,28^ SEM analysis showed MIL-89 to form fine spherical nanoparticles of a diameter of 76 ± 35 nm while MIL-89 PEG forms a more crystalline nanoparticle with an average diameter of 100 ± 38 nm (Fig. 1, Supplementary Fig. 1). The powder diffraction pattern for MIL-89 was indexed successfully in the hexagonal cell with *a* = 13.44(3) Å, *c* = 17.11(1) Å, and *V* = 2675 Å^3^ which is within the range of previously reported cell volumes^24^ for MIL-89 (1470–3900 Å^3^). IR/ATR for MIL-89 showed sharp peaks at 1600, 1370, 1000, and 850 *ν*(cm^–1^) while IR/ATR for MIL-89 PEG showed peaks at 1690, 1650, 1360, 1250, and 850 *ν*(cm^–1^). Thermogravimetric analysis showed both MIL-89 and MIL-89 PEG to be thermally stable up to at least 250℃ (Supplementary Fig. 1). At room temperature, MIL-89 PEG was stable for at least three months with no observable changes in the powder diffraction pattern over this time, while MIL-89 was less stable with observable loss of crystallinity after two months.

### Effect of MIL-89 and MIL-89 PEG on macrophage viability and iNOS activity

**Fig. 2.**
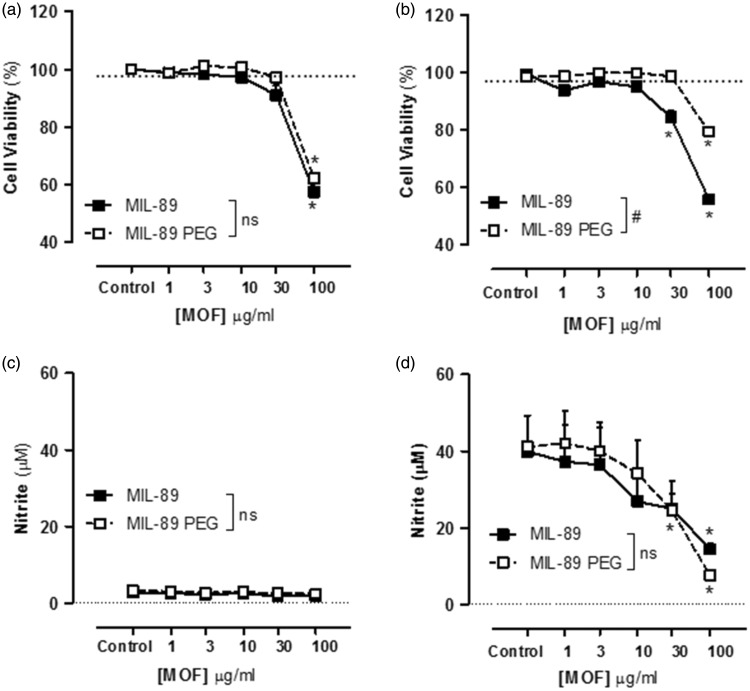
Effect of MIL-89 and MIL-89 PEG on J774 mouse macrophage cell viability under control (a) and inflammatory (LPS 1 µg/mL) (b) conditions and iNOS activity in the absence (c) and presence (d) of LPS. Data are mean ± SEM for n = 6 determinations; cells were treated for 24 h. Statistical analysis for effects between each MOF was determined by two-way ANOVA (#*P* < 0.05) and for each MOF compared to the relevant controls by one-way ANOVA followed by Dunnett’s Multiple Comparison Tests (*P < 0.05).

At concentrations of up to 10 µg/mL, neither MOF preparation affected cell viability (respiration), measured using AlamarBlue®, in murine macrophages under control culture conditions or when inflammation was induced by the addition of LPS (1 µg/mL) (Fig. 2). Both MOFs reduced macrophage viability at high concentrations with statistical significance noted for MIL-89 at 30 µg/mL and for MIL-89 PEG at 100 µg/mL (Fig. 2). Both MOFs inhibited LPS-induced iNOS activity in mouse macrophages. Whereas the effects seen with MIL-89 were in line with those on viability, the inhibitory effects of MIL-89 PEG at 30 µg/mL on iNOS activity were seen to be independent of changes in viability (Fig. 2). It should be noted that the starting materials used to make MIL-89 and MIL-89 PEG had no effect on cell viability or on iNOS activity (Supplementary Fig. 2).

### Effect of MIL-89 and MIL-89 PEG on endothelial cell viability, cell cytotoxicity, inflammatory response, and ET-1 release: comparison of cells from donors with or without PAH

**Fig. 3.**
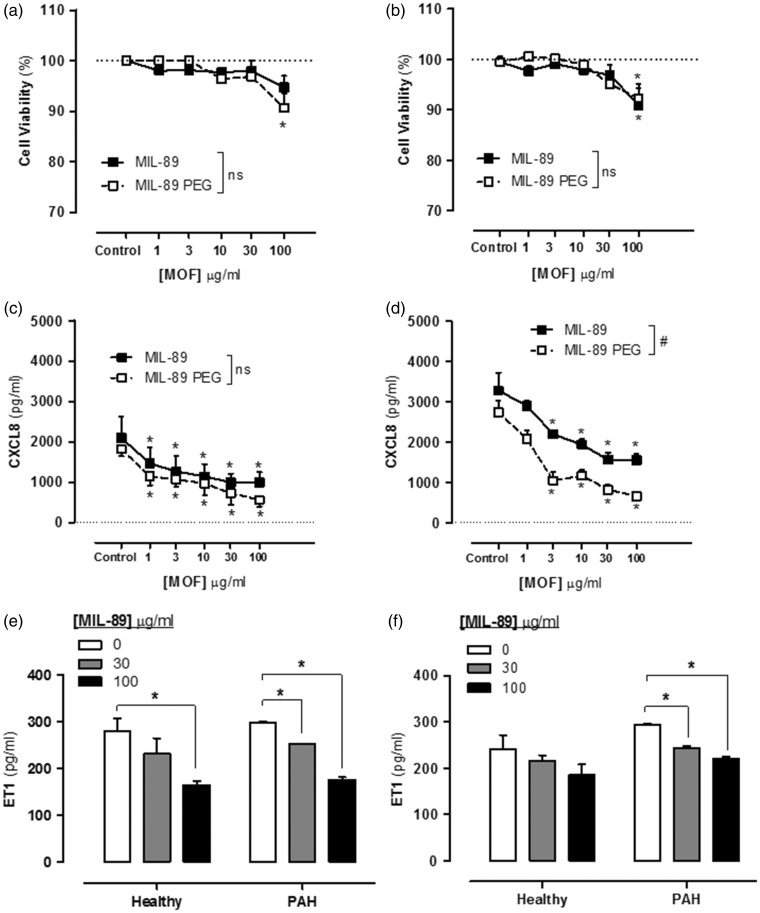
Effect of MIL-89 and MIL-89 PEG on endothelial cell viability (a, b), release of CXCL8 (c, d) and endothelin (ET)-1 (e, f). (a, c) Data from control donors (n = 6, three donors) and (b, d) from donors with PAH (n = 6 from three donors). Data are the mean ± SEM; cells were treated for 24 h. (a–d) Statistical analysis for effects between each MOF was determined by two-way ANOVA (#*P* < 0.05) and for each MOF compared to the relevant controls by one-way ANOVA followed by Dunnett’s Multiple Comparison Tests (*P < 0.05). (e, f) Data were analyzed using two-way ANOVA followed by Bonferroni post-tests (*P < 0.05).

At concentrations up to 30 µg/mL, neither MOF preparation affected viability (respiration), measured using AlamarBlue®, of endothelial cells from donors with or without PAH (Fig. 3). However, at very high concentrations (100 µg/mL), MOFs caused a small but statistically significant reduction in viability of cells from both groups of donors. Neither MIL-89 nor MIL-89 PEG induced cytotoxicity (measured by LDH release) at the concentrations tested (Supplementary Table 2). Similarly, MIL-89 preparations at concentrations up to 30 µg/mL did not affect cell number or nuclear shape (Supplementary Table 2). However, both preparations of MIL-89 inhibited the release of the inflammatory chemokine, CXCL8, in cells from both groups of donors, in a concentration dependent manner, with MIL-89 PEG being more potent than MIL-89 in cells from donors with PAH (Fig. 3). Importantly, both preparations of MOF inhibited release of ET-1 from endothelial cells, cultured from donors with or without PAH (Fig. 3).

### Effect of MIL-89 and MIL-89 PEG on PAVSMCs viability and inflammatory response

**Fig. 4.**
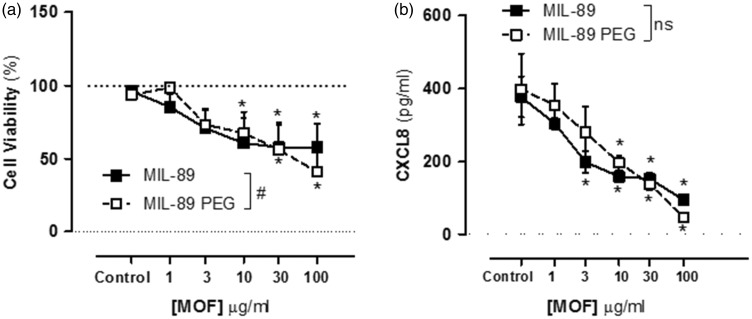
Effect of MIL-89 and MIL-89 PEG on pulmonary artery smooth muscle cell viability (a) and CXCL8 release (b). Data are mean ± SEM for n = 6 from three control donors; cells were treated for 24 h. Statistical analysis for effects between each MOF was determined by two-way ANOVA (#*P* < 0.05) and for each MOF compared to the relevant controls by one-way ANOVA followed by Dunnett’s multiple comparison post-test (*P < 0.05).

At concentrations up to 3 µg/mL, neither MOF preparation affected PAVSMCs viability. However, at higher concentrations (≥10 µg/mL) MIL-89 and MIL-89 PEG caused a concentration dependent reduction in cell viability and at 100 µg/mL cell number was reduced (Fig. 4, Supplementary Table 2). In addition, in PAVSMCs both preparations of MIL-89 inhibited the release of the inflammatory chemokine CXCL8 (Fig. 4).

### Effect of MIL-89 and MIL-89 PEG on PAECs, HASMCs, and A549 viability and inflammatory response

Neither MIL-89 nor MIL-89 PEG reduced PAEC viability at concentrations up to 3 µg/mL and did not cause a detectable cytotoxic effect or changes in nuclear shape despite a reduction in cell number at concentrations above 30 µg/mL (Supplementary Table 2). HASMCs were more sensitive to the effects of the MOFs which caused a reduction in viability and cell number at concentrations >30 µg/mL but without a noticeable change in the nuclear shape (Supplementary Table 2). In contrast, viability of human lung epithelial cells (A549) was not affected by either MIL-89 or MIL-89 PEG (Supplementary Table 2). Neither MIL-89 nor MIL-89 PEG caused apoptosis when measured using the Annexin V FITC staining method in any of the human lung cell lines studied (Supplementary Figs. 4 and 5).

### In vivo assessment of MIL-89

**Fig. 5.**
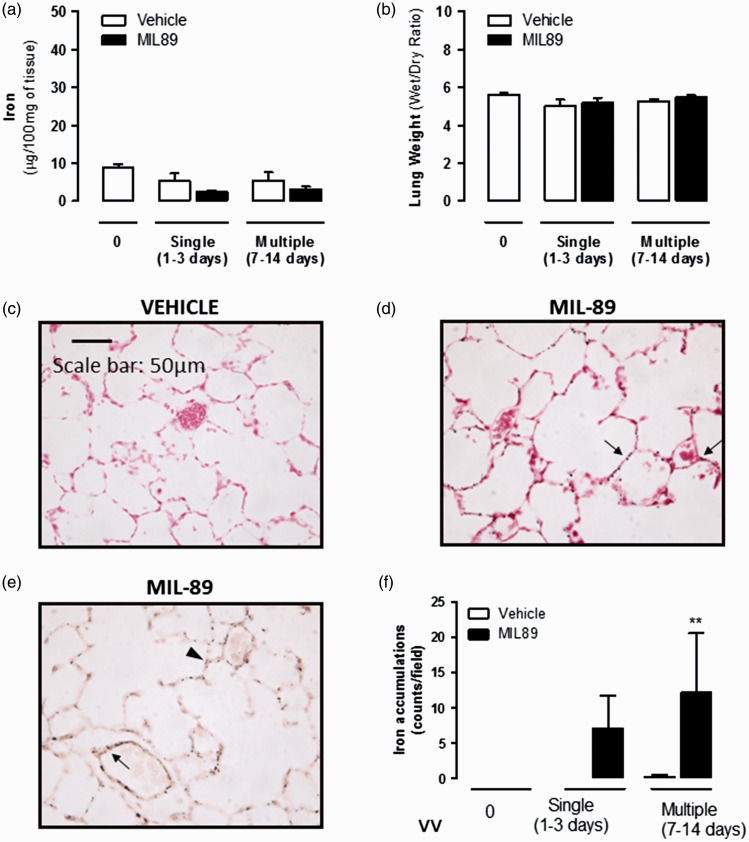
Effects of in vivo administration of MIL-89 on lung (a) gross total iron levels, (b) edema and (c–e) particle deposition. Effects of MIL-89 on iron levels in whole lung homogenates (a) and wet to dry lung weight ratio (b) following administration of single (1–3 days) or multiple (7–14 days) doses of MIL-89. (c, d) Representative images of lung sections stained with Prussian blue and annotated with arrows to show MIL-89 particles (×40; scale bar = 50 µm) from rats treated with vehicle or multiple dose of MIL-89. (e) Co-staining of the endothelial cell marker von Willebrand Factor (vWF; dark brown) and Prussian blue in lung sections from rats treated with MIL-89 for 14 days. The arrow denotes positive endothelial staining in a small pulmonary artery and the arrowhead in an alveolar capillary. (f) Quantification of the number of iron particles in the lungs from rats treated with MIL-89 with single (1–3 days) and multiple (7–14 days) doses are shown as the mean ± SEM from n = 4–6 rats at each time point. Statistical significance was analyzed by two-way ANOVA followed by Bonferroni post-test (**P < 0.01).

Administration of MIL-89 (50 mg/kg, twice a week) for up to two weeks did not affect total body weight (Supplementary Fig. 3) or notably affect animal behavior or condition. On the whole, gross iron levels were not increased in the plasma (Supplementary Table 3), lung (Fig. 5), or organs of animals treated with MIL-89. Levels of iron were increased in the spleen and the liver but only transiently and normalized after animals were treated with MIL-89 for 7–14 days (data not shown). However, measuring iron levels in tissues provides only a gross estimate and does not distinguish endogenous levels or specific state of the iron. By contrast to gross levels of iron, immunohistological analysis revealed detectable levels of the MOF particles within the lung (Fig. 5). As shown in Fig. 5e, immunostaining of the endothelial marker vWF revealed that iron particles accumulated mainly in alveolar capillaries although positive staining was also observed in small pulmonary arteries. Importantly, presence of MIL-89 in the lung had no effect on gross lung morphology and did not cause edema (Fig. 5). It should also be noted that particles were evident within the peritoneal cavity with an obvious presence in the great omentum consistent with excess MIL-89 accumulating at the site of injection (Supplementary Fig. 3).

MIL-89 did not cause oxidative stress or change the anti-oxidant capacity of plasma (Supplementary Table 3) or affect any of the following markers of organ failure or metabolic balance: urea, creatinine, total protein, albumin, globulin, albumin/globulin ratio (AG ratio), total bilirubin, cholesterol, glucose, calcium or inorganic phospholipids, alanine aminotransferase (ALT), alkaline phosphatase (ALK) (Supplementary Table 4). While MIL-89 did affect gamma-glutamyl transpeptidase, a marker of liver toxicity, when analyzed across multiple dosing (data not shown), it did produce a transient increase evident at day 7 that resolved by day 14 and was not seen throughout the rest of the dosing regime (Supplementary Figure 6).

## Discussion

PAH is a progressive and fatal disease that would benefit from a more targeted drug delivery approach which may limit side effects and potentially increase therapeutic exposure. In addition to vasodilator therapy, targeted drug delivery would enable cytostatic drugs such as Imatinib to be delivered directly to pulmonary vessels thereby evading the clinically limiting side effects associated with this drug.^41^ Here we have made and characterized nano-formulations of MOF MIL-89 to assess their suitability as carriers for drugs to treat PAH. MIL-89 and MIL-89 PEG were synthesized according to the procedure described previously by Horcajada et al.^24^ and structures were confirmed using a powder X-ray diffraction, SEM, and IR/TGA. Both MOFs (MIL-89 and MIL-89 PEG) displayed particulate sizes in the range of 50–150 nm, with the majority of the particles having a size of 100 ± 38 nm, which fits well with the original published report on these compounds.^24^ The predicted structure of MIL-89 is consistent with the literature where MIL-89 is able to shrink and close its pores when placed in dry environments^42^ while expanding and opening its pores when placed in an aqueous environment, a phenomenon often referred to as “breathing” (Fig. 1). The unit cell parameters (and hence pore size) for the MIL-89 MOF prepared in this work are similar to those reported for MIL-ht^27,42^ where the open form of MIL-89 was solvated with lutidine. PEGylating the MIL-89 improved uniformity of shape and also improved stability of the MOF material for up to three months at room temperature.

In their original publication, Horcajada et al. showed that MIL-89 was relatively non-toxic when assayed on mouse macrophages.^24^ Here we have performed similar protocols to assess the effects of the MOFs that we prepared on viability in the same cell type. As previously shown, MIL-89 had little or no effect on the viability of mouse macrophages at concentrations up to 10 µg/mL. In our study, we also tested the effect of MIL-89 PEG on mouse macrophages and found that the PEGylated form was less toxic in these cells. PAH is now thought of as an inflammatory condition. In order to better understand how MOFs might affect viability under inflammatory conditions, we tested the effects of MIL-89 and MIL-89 PEG in J774 mouse macrophages activated with LPS. Again, both MOFs were relatively non-toxic. J774 mouse macrophages release elevated levels of NO as a result of the inducible form of the enzyme that makes NO, iNOS, under inflammatory conditions, including those induced by LPS. Our data showed that both MIL-89 and MIL-89 PEG reduced iNOS activity, which in the case of MIL-89 was approximately in line with effects on viability. However, the effects of MIL-89 PEG on iNOS activity occurred at lower concentrations than those associated with reduced viability in the presence of LPS, which might suggest that this form of MOF has additional anti-inflammatory properties. The effects of MOFs on viability and on inflammatory responses were then explored in human lung cells including those directly relevant to PAH.

In PAH, endothelial cells and vascular smooth muscle become dysfunctional. Endothelial cells release increased constrictor mediators including ET-1 and inflammatory mediators including CXCL8. PASMCs, which are hyperproliferative in PAH, can also become activated and release inflammatory mediators, including CXCL8 in models of PAH. In this study, we took advantage of recent advances in stem cell technology that allow for endothelial cells to be successfully grown out from the blood of human donors, the so-called “blood outgrowth endothelial cells.”^43–46^ Our group^31^ and others^47^ have recently applied this technology to study endothelial cell phenotypes in patients with PAH. Neither MOF preparation, at concentrations up to 30 µg/mL, affected endothelial viability in cells from either control donors or from patients with PAH. Similar results were seen in PAECs where a reduction in cell viability was seen at concentrations >10 µg/mL without an increase in cell cytotoxicity at the tested concentrations suggesting that the reduction in cell viability is due to reduction in cell metabolism and not caused by cell death. As we have noted before, endothelial cells from PAH patients released increased levels of the inflammatory chemokine CXCL8, consistent with the idea that PAH has an inflammatory component. Both MOF preparation inhibited CXCL8 release by endothelial cells, an effect that was more prominent with MIL-89 PEG. Interestingly, as with CXCL8, both MOF preparations reduced ET-1 release, again MIL-89 PEG was more effective than authentic MIL-89, in endothelial cells from both control donors as well as from patients with PAH.

Whil MOFs had little effect on viability in endothelial cells, both MIL-89 and MIL-89 PEG induced cytotoxic effects in PASMCs with a reduction in cell viability and cell number and, at similar concentrations, release of CXCL8. Similar results were seen in HASMCs. In contrast, the lung epithelial cell line, A549 cells, were relatively insensitive to both MOF preparations.

The evaluation of the in vivo effects of MIL-89 revealed that this particular MOF is well tolerated, at least in the short term. Administration of MIL-89 for up to two weeks had no effect on body weight, lung edema, or plasma markers of organ failure and oxidative stress. However, increases in iron content in the spleen and the liver were found in this study, accompanied by a transient change in GGT, which returned to normal values by the end of the study. These data are in line with those reported by Horcajada et al.^24^ and suggest that the liver is the main place where MIL-89 is being cleared following i.p. administration. Importantly, histological analysis confirmed that MIL-89 was able to reach our target organ, the lung, without causing any detectable damage. Nonetheless, longer-term dosing and profiling of MIL-89 in a rodent PAH model is an essential progressive step before any application of this technology can be made in man.

In summary, we have successfully studied two MOF candidate materials with predicted cavity size compatible with the molecular sizes of drugs used to treat PAH. We have validated the structures and toxicity of these materials using methods described in the original publication by Horcajada et al.^24^ We went on to show that neither MIL-89 nor MIL-89 PEG is particularly toxic to endothelial cells, including those cultured from patients with PAH. However, the PEGylated form, MIL-89 PEG, was more stable and displayed anti-inflammatory effects at the level of CXCL8 and ET-1 release. In addition, in vascular smooth muscle cells, the MOFs induced toxicity. Since ET-1 is a therapeutic target in the treatment of PAH and because inhibition of vascular smooth muscle remodeling is a critical target to treat PAH, these properties, while at very high concentrations, could be considered as a therapeutic benefit. Finally, we have confirmed that MIL-89 is well-tolerated in the short term in vivo and accumulates in lung tissue. These findings form an essential part of the experimental process, further research now needs to be completed where PAH drugs are loaded into nanomedicine preparations and tested for toxicity and efficacy in longer-term and in PAH in vivo models. Our data suggest that MIL-89, and particularly MIL-89 PEG, should be considered as suitable candidates that would not only provide a drug carrier but also provide added therapeutic benefit.^48^ This work serves as a first step in the translation to a new drug formulation for PAH. Now knowing that MOFs such as MIL-89 are viable starting points the next steps would be to load the structures with a drug and to devise a specific delivery strategy to affected pulmonary vessels. One approach to this would be to use an antibody-drug conjugate. Here it would first be necessary to identify a specific antigen expressed locally within pulmonary vessels and manufacture and humanize the antibody, another possible targeting scheme is by using the homing peptide CAR as described previously.^49^ These steps remain the subject of ongoing research.

## Supplementary Material

Supplementary material

## References

[1] Montani D Gunther S Dorfmuller P et al. Pulmonary arterial hypertension. Orphanet J Rare Dis 2013; 8(1): 97. 23829793 10.1186/1750-1172-8-97 PMC3750932

[2] Wagenvoort CA Wagenvoort N Primary Pulmonary Hypertension, New York: Wiley & Sons, 1977.

[3] Simonneau G, Gatzoulis MA, Adatia I, et al. Updated clinical classification of pulmonary hypertension. *J Am Coll Cardiol* 2013; 62(25 Suppl): D34–41. 10.1016/j.jacc.2013.10.029 24355639

[4] Archer SL Weir EK Wilkins MR Basic science of pulmonary arterial hypertension for clinicians: new concepts and experimental therapies. Circulation 2010; 121(18): 2045–2066. 20458021 10.1161/CIRCULATIONAHA.108.847707 PMC2869481

[5] George PM Oliver E Dorfmuller P et al. Evidence for the involvement of type I interferon in pulmonary arterial hypertension. Circ Res 2014; 114(4): 677–688. 24334027 10.1161/CIRCRESAHA.114.302221 PMC4006084

[6] Budhiraja R Tuder RM Hassoun PM Endothelial dysfunction in pulmonary hypertension. Circulation 2004; 109(2): 159–165. 14734504 10.1161/01.CIR.0000102381.57477.50

[7] Galie N Manes A Branzi A Medical therapy of pulmonary hypertension. The prostacyclins. *Clin Chest Med* 2001; 22(3): 529–537, x. 11590846 10.1016/s0272-5231(05)70289-6

[8] Christman BW McPherson CD Newman JH et al. An imbalance between the excretion of thromboxane and prostacyclin metabolites in pulmonary hypertension. N Engl J Med 1992; 327(2): 70–75. 1603138 10.1056/NEJM199207093270202

[9] McGoon MD Kane GC Pulmonary hypertension: diagnosis and management. Mayo Clin Proc 2009; 84(2): 191–207. 19181654 10.4065/84.2.191 PMC2664591

[10] Waxman AB Zamanian RT Pulmonary arterial hypertension: new insights into the optimal role of current and emerging prostacyclin therapies. Am J Cardiol 2013; 111(5 Suppl): 1A–16A. 23414683 10.1016/j.amjcard.2012.12.002

[11] Jeffery TK Morrell NW Molecular and cellular basis of pulmonary vascular remodeling in pulmonary hypertension. Prog Cardiovasc Dis 2002; 45(3): 173–202. 12525995 10.1053/pcad.2002.130041

[12] Giaid A Yanagisawa M Langleben D et al. Expression of endothelin-1 in the lungs of patients with pulmonary hypertension. N Engl J Med 1993; 328(24): 1732–1739. 8497283 10.1056/NEJM199306173282402

[13] Rickert V Haefeli WE Weiss J Pharmacokinetic interaction profile of riociguat, a new soluble guanylate cyclase stimulator, in vitro. Pulm Pharmacol Ther 2014; 28(2): 130–137. 24657506 10.1016/j.pupt.2014.02.004

[14] Sebkhi A Strange JW Phillips SC et al. Phosphodiesterase type 5 as a target for the treatment of hypoxia-induced pulmonary hypertension. Circulation 2003; 107(25): 3230–3235. 12796132 10.1161/01.CIR.0000074226.20466.B1

[15] Mitchell JA Ahmetaj-Shala B Kirkby NS et al. Role of prostacyclin in pulmonary hypertension. Glob Cardiol Sci Pract 2014; 2014(4): 382–393. 25780793 10.5339/gcsp.2014.53 PMC4355513

[16] Brenner JS Greineder C Shuvaev V et al. Endothelial nanomedicine for the treatment of pulmonary disease. Expert Opin Drug Deliv 2015; 12(2): 239–261. 25394760 10.1517/17425247.2015.961418 PMC8135185

[17] Roco MC Nanotechnology: convergence with modern biology and medicine. Curr Opin Biotechnol 2003; 14(3): 337–346. 12849790 10.1016/s0958-1669(03)00068-5

[18] Rosler A Vandermeulen GW Klok HA Advanced drug delivery devices via self-assembly of amphiphilic block copolymers. Adv Drug Deliv Rev 2001; 53(1): 95–108. 11733119 10.1016/s0169-409x(01)00222-8

[19] Mosgoeller W Prassl R Zimmer A Nanoparticle-mediated treatment of pulmonary arterial hypertension. Methods Enzymol 2012; 508: 325–354. 22449934 10.1016/B978-0-12-391860-4.00017-3

[20] Mohamed NA Ahmetaj-Shala B Duluc L et al. A new NO-releasing nanoformulation for the treatment of pulmonary arterial hypertension. J Cardiovasc Transl Res 2016; 9(2): 162–164. 26960567 10.1007/s12265-016-9684-2 PMC4830862

[21] Keskin S Kızılel S Biomedical applications of metal organic frameworks. Ind Eng Chem Res 2011; 50(4): 1799–1812.

[22] Ferey G Mellot-Draznieks C Serre C et al. A chromium terephthalate-based solid with unusually large pore volumes and surface area. Science 2005; 309(5743): 2040–2042. 16179475 10.1126/science.1116275

[23] Horcajada P Serre C Vallet-Regi M et al. Metal-organic frameworks as efficient materials for drug delivery. Angew Chem Int Ed Engl 2006; 45(36): 5974–5978. 16897793 10.1002/anie.200601878

[24] Horcajada P Chalati T Serre C et al. Porous metal-organic-framework nanoscale carriers as a potential platform for drug delivery and imaging. Nat Mater 2010; 9(2): 172–178. 20010827 10.1038/nmat2608

[25] Alkordi MH Self-assembled metal-organic polyhedra (MOPs): opportunities in biomedical applications. Glob Cardiol Sci Pract 2013; 2013(1): 37–43. 24689000 10.5339/gcsp.2013.6 PMC3963725

[26] Huxford RC Della Rocca J Lin W Metal-organic frameworks as potential drug carriers. Curr Opin Chem Biol 2010; 14(2): 262–268. 20071210 10.1016/j.cbpa.2009.12.012 PMC2847625

[27] Serre C Surble S Mellot-Draznieks C et al. Evidence of flexibility in the nanoporous iron(iii) carboxylate MIL-89. Dalton Trans 2008; 40: 5462–5464. 10.1039/b805408h 19082028

[28] Surble S Millange F Serre C et al. An EXAFS study of the formation of a nanoporous metal-organic framework: evidence for the retention of secondary building units during synthesis. Chem Commun (Camb) 2006; 14: 1518–1520. 10.1039/b600709k 16575446

[29] Schneider CA Rasband WS Eliceiri KW NIH Image to ImageJ: 25 years of image analysis. Nat Methods 2012; 9(7): 671–675. 22930834 10.1038/nmeth.2089 PMC5554542

[30] Starke RD Paschalaki KE Dyer CE et al. Cellular and molecular basis of von Willebrand disease: studies on blood outgrowth endothelial cells. Blood 2013; 121(14): 2773–2784. 23355534 10.1182/blood-2012-06-435727 PMC3617637

[31] George PM Oliver E Dorfmuller P et al. Evidence for the involvement of type I interferon in pulmonary arterial hypertension. Circ Res 2014; 114(4): 677–688. 24334027 10.1161/CIRCRESAHA.114.302221 PMC4006084

[32] Belvisi MG Saunders M Yacoub M et al. Expression of cyclo-oxygenase-2 in human airway smooth muscle is associated with profound reductions in cell growth. Br J Pharmacol 1998; 125(5): 1102–1108. 9846651 10.1038/sj.bjp.0702104 PMC1565660

[33] Mitchell JA Belvisi MG Akarasereenont P et al. Induction of cyclo-oxygenase-2 by cytokines in human pulmonary epithelial cells: regulation by dexamethasone. Br J Pharmacol 1994; 113(3): 1008–1014. 7858842 10.1111/j.1476-5381.1994.tb17093.x PMC1510466

[34] Moreno L McMaster SK Gatheral T et al. Nucleotide oligomerization domain 1 is a dominant pathway for NOS2 induction in vascular smooth muscle cells: comparison with Toll-like receptor 4 responses in macrophages. Br J Pharmacol 2010; 160(8): 1997–2007. 20649597 10.1111/j.1476-5381.2010.00814.x PMC2913099

[35] Gatheral T Reed DM Moreno L et al. A key role for the endothelium in NOD1 mediated vascular inflammation: comparison to TLR4 responses. PLoS One 2012; 7(8): e42386. 22870324 10.1371/journal.pone.0042386 PMC3411636

[36] Baati T Njim L Neffati F et al. In depth analysis of the in vivo toxicity of nanoparticles of porous iron(III) metal-organic frameworks. Chem Sci 2013; 4(4): 1597–1607.

[37] Jung C Kaul MG Bruns OT et al. Intraperitoneal injection improves the uptake of nanoparticle-labeled high-density lipoprotein to atherosclerotic plaques compared with intravenous injection: a multimodal imaging study in ApoE knockout mice. Circ Cardiovasc Imaging 2014; 7(2): 303–311. 24357264 10.1161/CIRCIMAGING.113.000607

[38] Simon-Yarza T Baati T Neffati F et al. In vivo behavior of MIL-100 nanoparticles at early times after intravenous administration. Int J Pharm 2016; 511(2): 1042–1047. 27515292 10.1016/j.ijpharm.2016.08.010

[39] Baati T Njim L Neffati F et al. In depth analysis of the in vivo toxicity of nanoparticles of porous iron(III) metal–organic frameworks. Chem Sci 2013; 4: 1597–1607.

[40] Moral-Sanz J Lopez-Lopez JG Menendez C et al. Different patterns of pulmonary vascular disease induced by type 1 diabetes and moderate hypoxia in rats. Exp Physiol 2012; 97(5): 676–86. 22247283 10.1113/expphysiol.2011.062257

[41] Frost AE Barst RJ Hoeper MM et al. Long-term safety and efficacy of imatinib in pulmonary arterial hypertension. J Heart Lung Transplant 2015; 34(11): 1366–1375. 26210752 10.1016/j.healun.2015.05.025

[42] Serre C Millange F Surble S et al. A route to the synthesis of trivalent transition-metal porous carboxylates with trimeric secondary building units. Angew Chem Int Ed Engl 2004; 43(46): 6285–6289. 15372643 10.1002/anie.200454250

[43] Martin-Ramirez J Hofman M van den Biggelaar M et al. Establishment of outgrowth endothelial cells from peripheral blood. Nat Protoc 2012; 7(9): 1709–1715. 22918388 10.1038/nprot.2012.093

[44] Ingram DA Mead LE Tanaka H et al. Identification of a novel hierarchy of endothelial progenitor cells using human peripheral and umbilical cord blood. Blood 2004; 104(9): 2752–2760. 15226175 10.1182/blood-2004-04-1396

[45] Starke RD Ferraro F Paschalaki KE et al. Endothelial von Willebrand factor regulates angiogenesis. Blood 2011; 117(3): 1071–1080. 21048155 10.1182/blood-2010-01-264507 PMC3035068

[46] Reed DM Paschalaki KE Starke RD et al. An autologous endothelial cell:peripheral blood mononuclear cell assay that detects cytokine storm responses to biologics. FASEB J 2015; 29(6): 2595–2602. 25746794 10.1096/fj.14-268144

[47] Lavoie JR Ormiston M Perez-Iratxeta C et al. Proteomic analysis implicates translationally controlled tumor protein as a novel mediator of occlusive vascular remodeling in pulmonary arterial hypertension. Circulation 2014; 129(21): 2125–2135. 24657995 10.1161/CIRCULATIONAHA.114.008777

[48] Della Rocca J Liu D Lin W Nanoscale metal-organic frameworks for biomedical imaging and drug delivery. Acc Chem Res 2011; 44(10): 957–968. 21648429 10.1021/ar200028a PMC3777245

[49] Toba M Alzoubi A O’Neill K et al. A novel vascular homing peptide strategy to selectively enhance pulmonary drug efficacy in pulmonary arterial hypertension. Am J Pathol 2014; 184(2): 369–375. 24401613 10.1016/j.ajpath.2013.10.008 PMC3906494

