# Supplementary material for: Chemical and biological assessment of metal organic frameworks (MOFs) in pulmonary cells and in an acute in vivo model: relevance to pulmonary arterial hypertension therapy
Source: Pulm Circ. 2017 Jun 27;7(3):643–53. doi: 10.1177/2045893217710224 (PMC5841901; doi:10.1177/2045893217710224)
Supplement: Supplementary material [file PUL710224_supplementary_figures.pdf]

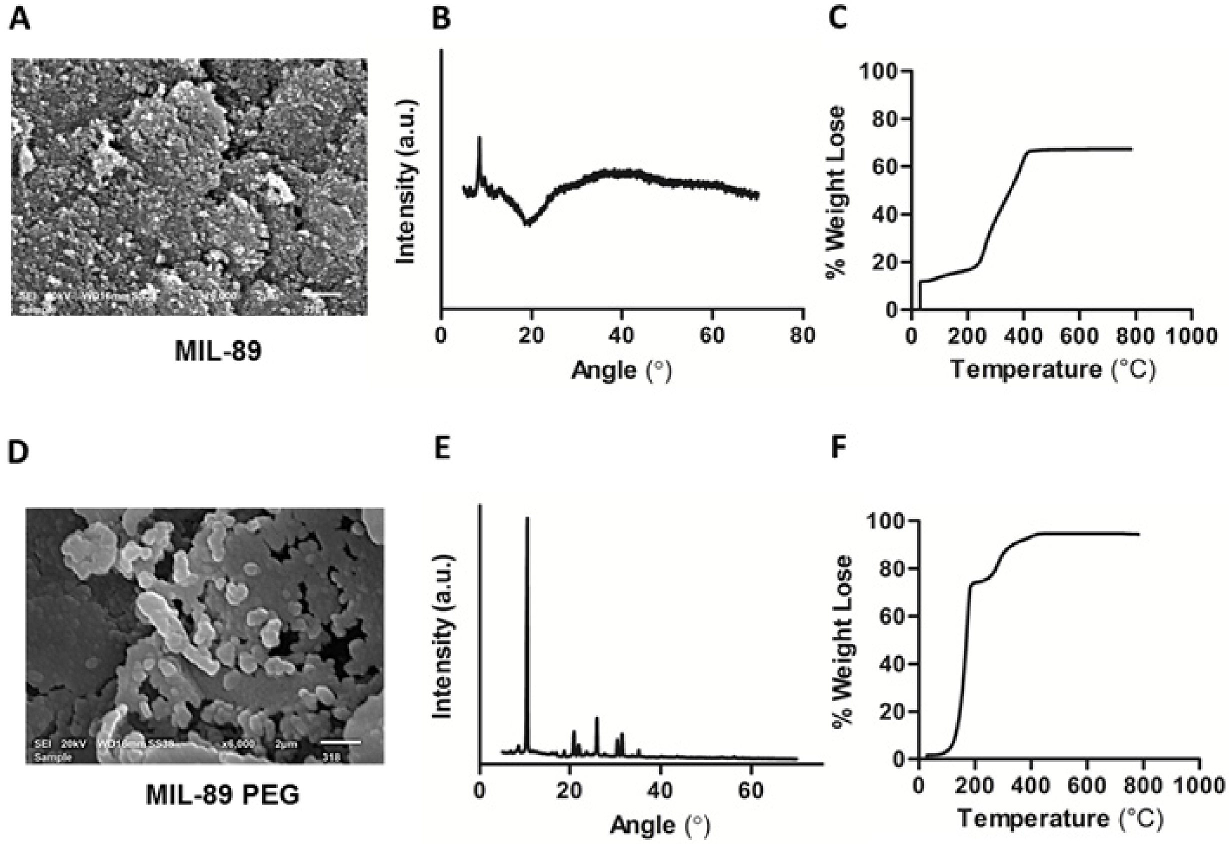

Supplementary Figure 1:

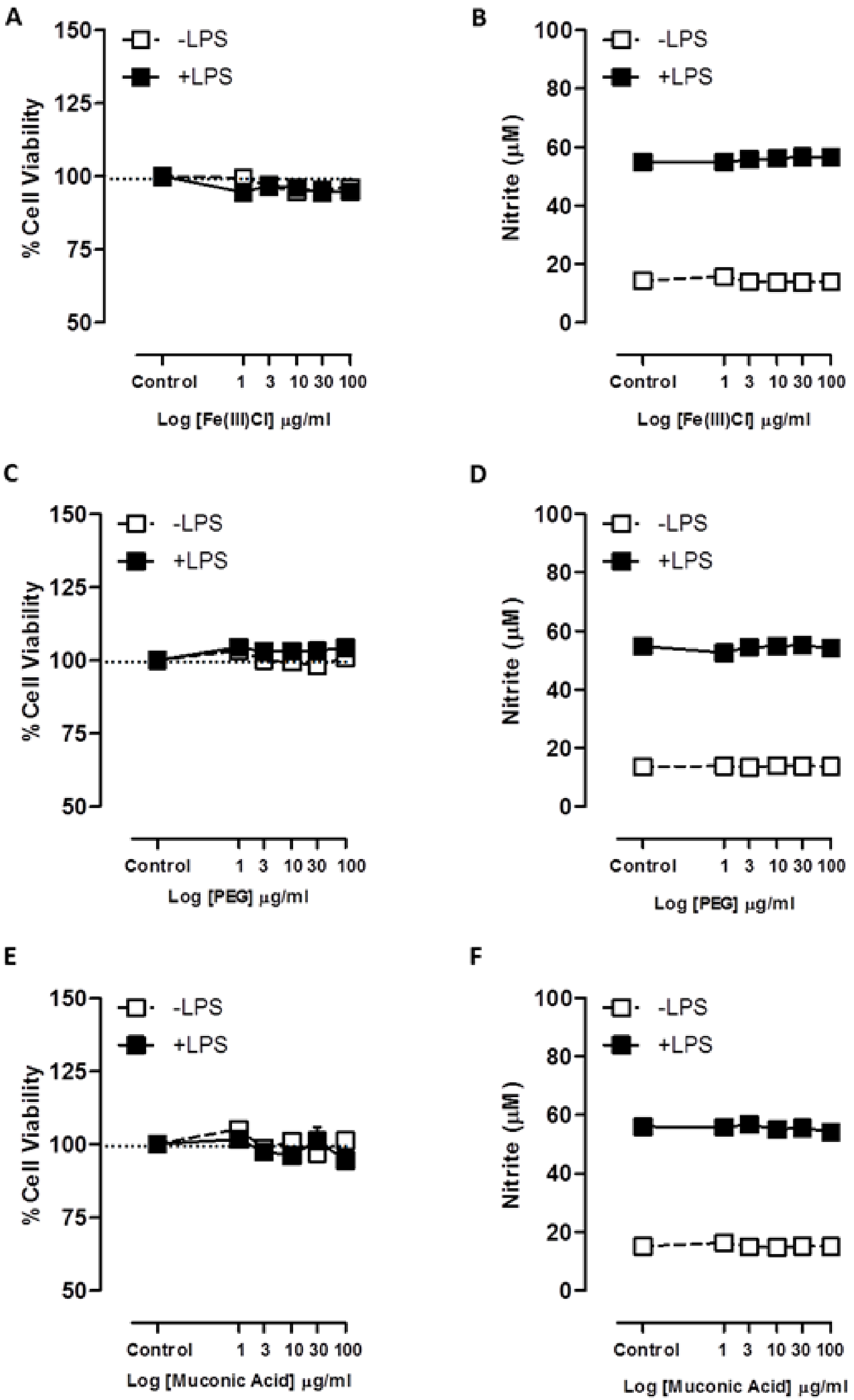

Supplementary Figure2

A

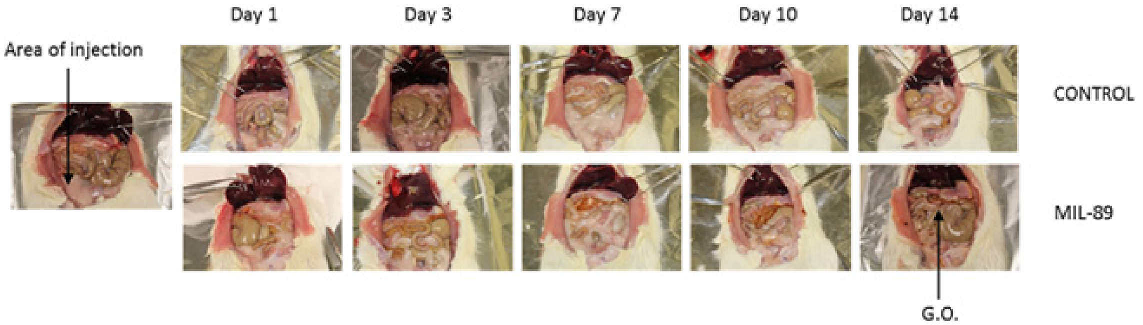

B

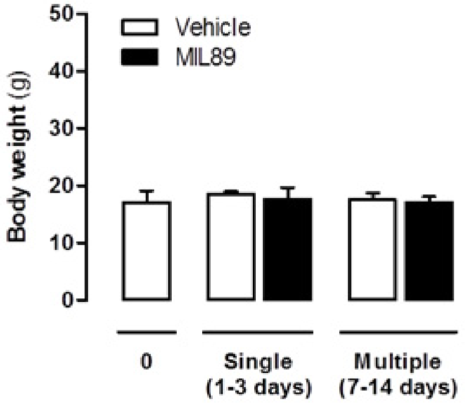

Supplementary Figure 3:

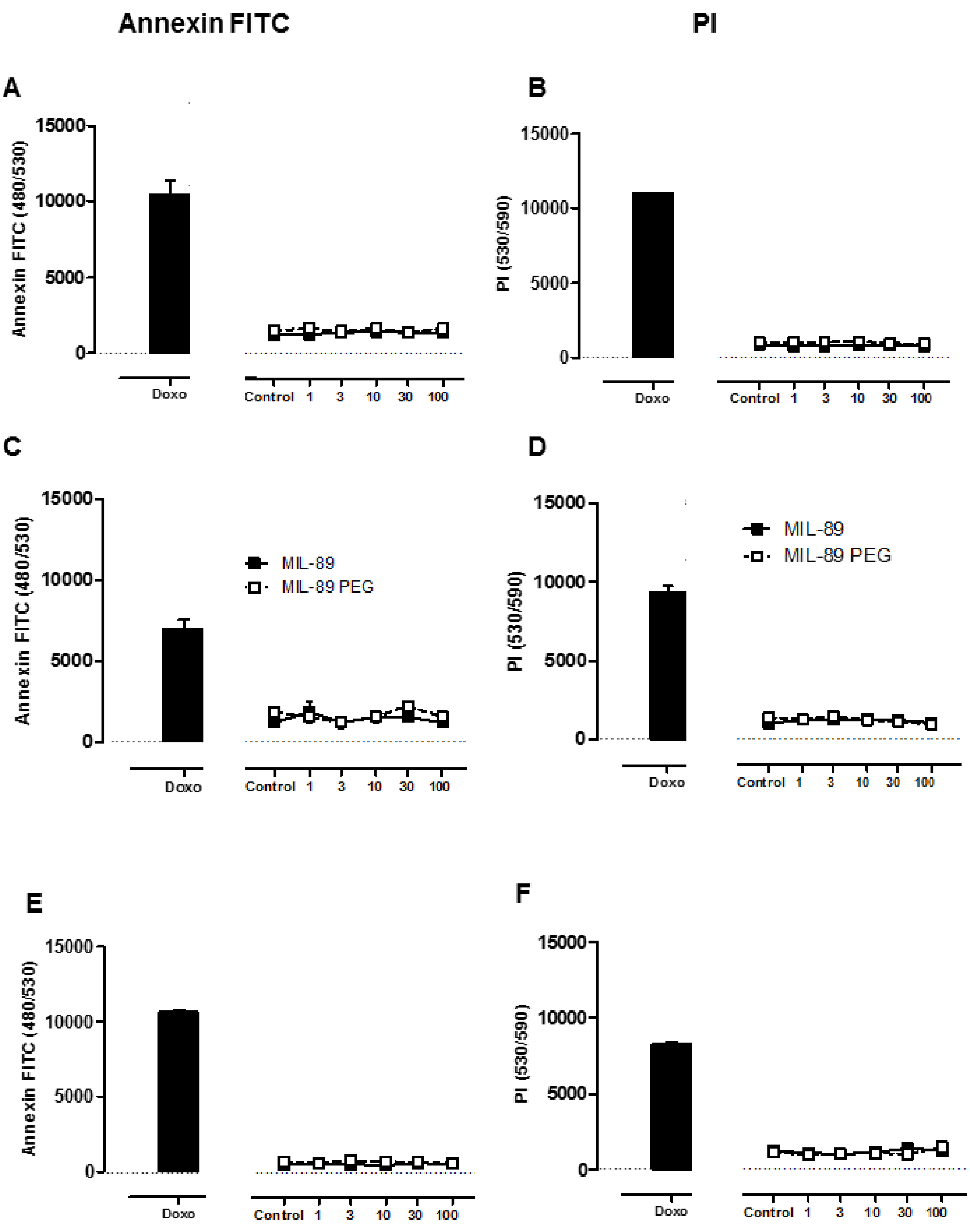

Supplementary Figure 4:

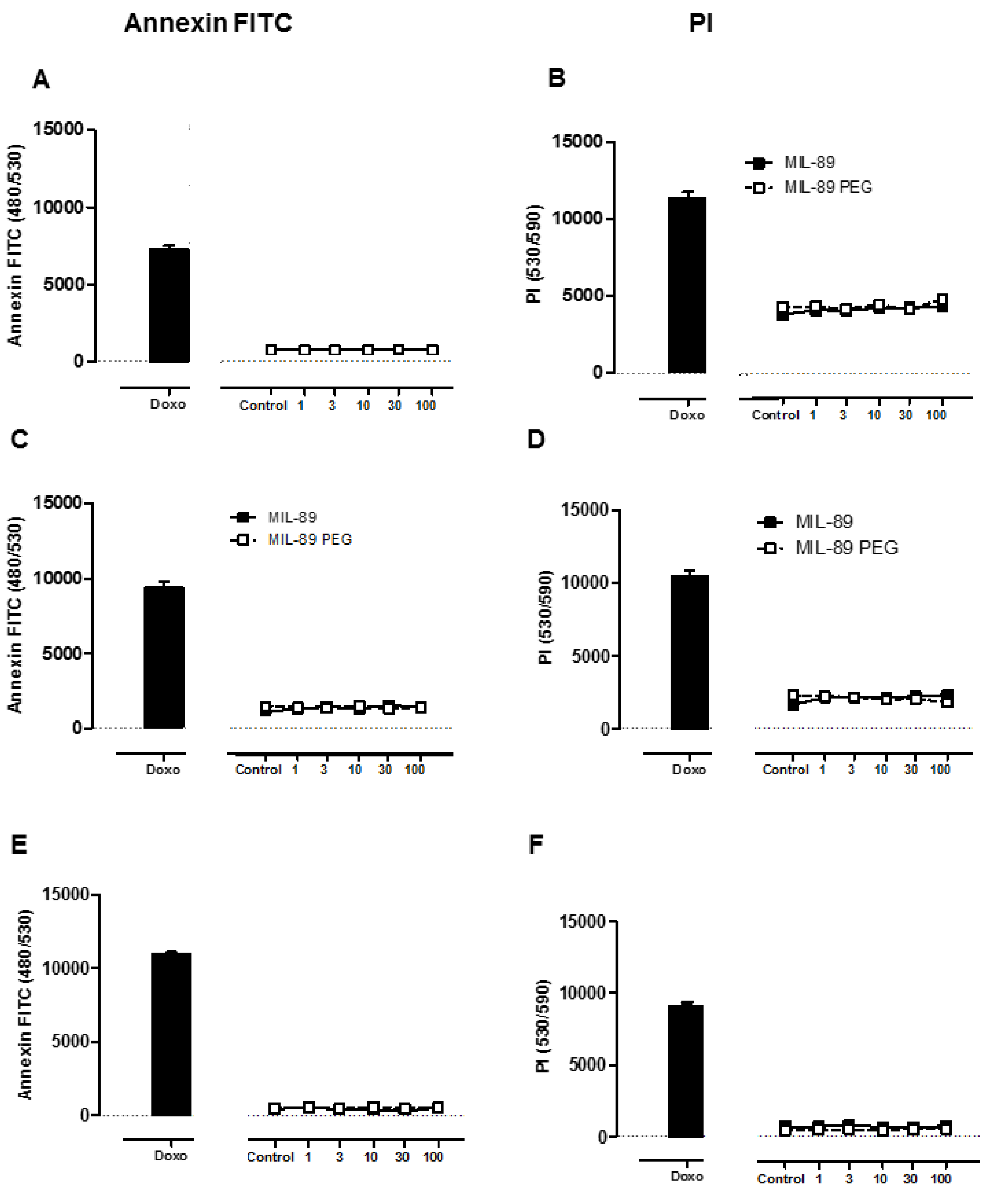

Supplementary Figure 5:

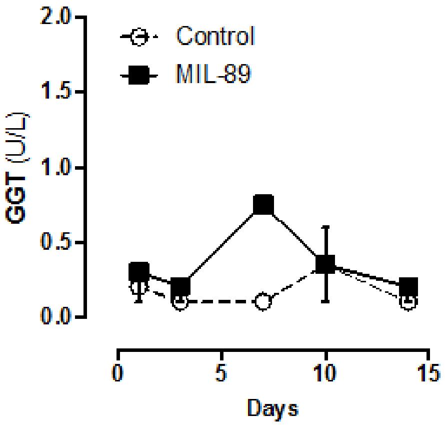

Supplementary Figure 6:
